# Le Programme d'Appui aux Stratégies Sociales (PASS), une poignée de mains entre mutualistes du Nord et du Sud (Europe-Afrique)

**DOI:** 10.48327/mtsimagazine.n1.2021.94

**Published:** 2021-04-28

**Authors:** J.-V. Ayité

**Affiliations:** Directeur Général, Programme d'Appui aux Stratégies Sociales (PASS)

**Keywords:** Mutualité, Mutualiste, Mutuelle, Afrique, Financement de la santé, Protection sociale, Mutuality, Mutualist, Mutual insurance, Africa, Health financing, Social protection

## Abstract

Le Programme d'Appui aux Stratégies Sociales, (PASS), est un pôle d'expertise en santé et protection sociale à but non lucratif qui prolonge les activités du Programme d'appui aux stratégies mutualistes de santé, géré par Expertise France de 2015 à 2020. La structure est opérationnelle depuis août 2019 grâce au soutien de deux groupements mutualistes français, le Groupe Vyv et la Fédération Nationale de la Mutualité Française (FNMF), sous la forme d'une société par actions simplifiées à but non lucratif. Partant de son socle originel d'appui au développement des couvertures santé mutualistes, le PASS élargit son domaine d'intervention aux projets, politiques et systèmes de santé et de protection sociale avec une attention particulière sur les problématiques liées aux femmes et aux enfants. Le PASS propose ainsi une expertise construite autour de 3 domaines d'activité stratégiques qui sont: (1) l'appui aux mécanismes solidaires de financement de la santé de type mutualiste (audit, études, conseil, formation); (2) l'appui au renforcement des services de santé bâtis en lien avec les politiques publiques et (3) l'apport en expertise et en capacités opérationnelles en réponse à diverses problématiques ponctuelles de santé et de protection sociale. Basé à Abidjan en Côte d'Ivoire, le PASS étend l'ensemble de ses activités aux autres pays d'Afrique francophone.

## Abstract (en)

The Programme d'Appui aux Stratégies Sociales, (PASS), is a non-profit health and social protection expertise organization that extends the activities of the Programme d'Appui aux Stratégies Mutuelles de Santé, managed by Expertise France from 2015 to 2020. The structure has been operational since August 2019 thanks to the support of two French mutualist groups, the Vyv Group and the Fédération Nationale de la Mutualité Française (FNMF), in the form of a non-profit simplified joint stock company. Starting from its original base of support for the development of mutual health coverage, PASS has broadened its field of intervention to include health and social protection projects, policies and systems, with a particular focus on issues related to women and children. PASS thus proposes an expertise built around 3 strategic areas of activity which are: (1) support for mutual health financing mechanisms (audit, studies, consulting, training); (2) support for the strengthening of health services built in conjunction with public policies; and (3) provision of expertise and operational capacities in response to various health and social protection issues. Based in Abidjan, Côte d'Ivoire, PASS is extending its activities to other French-speaking African countries.

Une large part des populations d'Afrique subsaharienne vit actuellement dans un contexte d'extrême pauvreté et souffre de graves problèmes sanitaires^1^. Depuis l'Initiative de Bamako en 1987, les États se sont retirés progressivement de la subvention massive de l'accès aux soins, de sorte qu'aujourd'hui, les usagers doivent y participer financièrement dans une logique de recouvrement des coûts. Ce retrait du secteur public dans le financement de la santé a eu d'importantes conséquences pour les populations, en particulier celles du secteur informel. En outre, l'insuffisance ou l'inaccessibilité des marchés assurantiels privés empêchent nombre d'individus de bénéficier d'une couverture contre le risque maladie. Privées de protection sociale, ces populations se voient souvent dans l'incapacité financière d'accéder à des soins de santé de qualité. Ce contexte a favorisé l’émergence des mutuelles de santé en Afrique. Leur principal objectif étant d'améliorer l'accès des populations aux soins de santé. La pertinence de leur rôle a été réaffirmé dans le rapport présenté par l'Association Internationale de la Sécurité Sociale (AISS) lors du forum mondial de la sécurité sociale en 2013 à Doha. L'AISS a reconnu le modèle mutualiste comme « un outil pour le développement de la protection sociale dans le monde et en particulier dans le secteur de la santé. ». C'est à partir de là et de réflexions menées avec d'autres coupoles internationales, que l'idée du Programme d'Appui aux Stratégies mutualistes de Santé (PASS) s'est concrétisée avec l'appui d'acteurs mutualistes européens et Africains. Il s'est mis en place dans la zone de l'Union Économique et Monétaire Ouest Africaine (UEMOA), où une réglementation commune de la mutualité sociale a été adoptée par les différents gouvernements et où la majorité des pays se sont lancés dans des politiques de couverture sanitaire universelle.

Conscients de ce que les mutuelles de santé peuvent apporter à leur pays, et afin de prévenir toutes sortes de dérapage, les pays membres de l'UEMOA ont adopté le Règlement n°07/2009/CM/UEMOA du 26 juin 2009 portant réglementation de la mutualité sociale au sein de l'UEMOA^2^.

Cette initiative des États a fait de la zone UEMOA la première dotée d'un cadre juridique pour la mutualité, et par la même occasion le point idéal pour renforcer le mouvement mutualiste en Afrique.

En août 2016, la Banque Mondiale, avec l'Organisation Mondiale de la Santé, le gouvernement du Japon, l'Agence Japonaise de Coopération Internationale, le Fonds mondial de lutte contre le sida, la tuberculose et le paludisme (Fonds mondial) et la Banque Africaine de Développement (BAD) ont lancé un document fondateur: la CSU en Afrique: un cadre d'action^3^. Le document estime que l'investissement dans les systèmes de santé africains est la clé d'une croissance inclusive et durable. Malgré la forte croissance économique de ces dernières années qui a contribué à réduire la pauvreté à 43 % de la population^4^, de nombreux pays font encore face à des taux élevés de mortalité infantile et maternelle, et la plupart des systèmes de santé ne sont pas en mesure de faire face efficacement aux épidémies et au fardeau croissant des maladies chroniques. Ainsi, cinq des huit États membres de l'UEMOA se sont appropriés ce cadre et se sont lancés dans des politiques de CSU adaptées à leur contexte. Mais les défis restent encore nombreux. Il faut définir et étendre des services garantis, développer des systèmes de financement de la santé pour financer les services de santé et assurer une protection financière, une disponibilité de service de qualité, améliorer la gouvernance et la gestion et prendre toutes autres mesures de renforcement des systèmes de santé.

## Qu'est-ce que le PASS, en plus d’être …

Comme prévu au démarrage du Programme d'appui aux stratégies mutualistes de santé (PASS-1) et dans une démarche innovante, le programme s'est aujourd'hui transformé en une structure pérenne et autofinancée en capacité de poursuivre son action de manière autonome.

Dans le cadre de son activité, le programme s'attelle à apporter son expertise au mouvement mutualiste africain. Cependant, ses activités vont désormais au-delà de l'appui au développement des couvertures santé mutualistes et se regroupent autour de trois domaines d'intervention:

- L'appui aux mécanismes solidaires de financement de la santé de types mutualistes;
- L'appui au renforcement des services de santé et de protection sociale bâtis en lien avec les politiques publiques;
- L'apport en expertise et en capacités opérationnelles en réponse à diverses problématiques ponctuelles de santé et de protection sociale.

Le PASS est composé d'une équipe engagée à contribuer à l'amélioration du bien-être des populations sur le continent africain, en vue d'un meilleur accès à la santé et aux filets de protection sociale à des coûts abordables quand cela est possible, ou sans contrepartie lorsqu'il s'agit de communautés et groupes en situation de vulnérabilité sévère.

## … un outil pour la création et le développement des mutuelles, …

Ce programme aide les mutuelles des pays de l'UEMOA à être un pilier incontournable dans le cadre de divers projets d'extension de la couverture santé. Le PASS appuie les acteurs mutualistes des huit pays de la zone UEMOA dans la définition de leurs objectifs et la mise en œuvre de leurs stratégies. Les dirigeants de plus d'une centaine de mutuelles de santé ont été formés, notamment sur les thématiques d'initiation à la protection sociale, à la couverture santé universelle, à la mutualité, à la gouvernance, à la gestion administrative et financière des mutuelles, pour ne citer que ces modules.

Le PASS est également engagé sur la question du renforcement du leadership des femmes dans la gouvernance des mécanismes de couverture santé solidaire. En 2019 et 2020, le programme a organisé « la journée internationale de la femme mutualiste », un forum d’échanges entre dirigeantes de structures mutualistes et de l’économie sociale en général du Sud et du Nord.

Ces rencontres ont permis de créer le Réseau International des Femmes Solidaires qui porte des projets, allant du renforcement des capacités des femmes dirigeantes notamment sur le leadership, au plaidoyer institutionnel, pour une meilleure représentativité des femmes aux postes de décision dans les structures de l’économie sociale et solidaire.

Au-delà du renforcement des capacités de ces mutuelles, le PASS se penche sur le renforcement des systèmes de santé et de protection sociale, en adoptant une approche qui prend en compte le financement, la prestation de services et la gouvernance des politiques.

Le PASS a travaillé sur plusieurs projets de développement de mutuelles allant de la fusion de portefeuille de santé, aux études préliminaires de construction d'une offre de soins.

Plusieurs projets de construction de mécanisme de prise en charge du risque santé ont été envisagés et mis en œuvre avec le secteur informel (artisans, agriculteurs…).

Notons que le PASS accompagne également, à leur demande, les structures étatiques et les institutions régionales dans la mise en œuvre des politiques de développement de la mutualité ou de couverture santé universelle.

Ainsi, en tant que membre invité permanent, le PASS appuie le Comité Consultatif de la Mutualité Sociale de l'UEMOA dans ses missions. Il accompagne les deux agences de régulation de la mutualité de la zone UEMOA (au Niger et en Côte d'Ivoire). Le programme appuie également les structures créées par les États pour piloter les politiques de couverture santé universelle notamment sur les questions liées au secteur informel qui à lui seul représente plus de 80 % des populations de la zone.

## … acteur de synergies …

Le PASS fait sienne la politique des soins de santé primaires (SSP) et manifeste son attachement aux valeurs et aux principes sur lesquels reposent les SSP: l’équité, la justice sociale, la participation, et la collaboration intersectorielle. L'intérêt pour le PASS d'intégrer les dimensions relatives à la promotion de la santé dans ses projets est de le positionner comme un acteur qui accompagne les populations dans l'amélioration de la maîtrise de leur propre santé. La promotion de la santé couvre une vaste gamme d'interventions sociales et environnementales conçues pour favoriser et protéger la santé et la qualité de vie au niveau individuel en luttant contre les principales causes de la mauvaise santé, notamment par la prévention, et en ne s'intéressant pas seulement au traitement et à la guérison.

## … pour un meilleur accès à la santé et aux filets de protection sociale en Afrique.

Le PASS se positionne désormais en tant que pôle d'expertise de niveau international, capable d'apporter aux ministères en charge des politiques de la santé et aux ministères en charge des politiques de protection sociale de la région Afrique, les avis et les conseils en matière de renforcement des systèmes de santé (RSS) et des socles de protection sociale. Ce pôle d'expertise se mobilise également sur la prospective, indispensable pour éclairer les décisions ayant des effets structurants à long terme. Le projet de mise en place d'un observatoire de la santé et de la protection sociale est en cours de développement et le réseau d'experts du PASS est constamment consolidé pour conduire les études nécessaires et animer les dialogues politiques indispensables à cet axe stratégique.

Comme on peut le constater, en progressant au-delà du champ mutualiste, c'est l'ensemble du pilier lié au financement de la santé que le PASS embrasse désormais en proposant des solutions techniques et des arrangements institutionnels susceptibles d'exercer des effets positifs sur l'offre de soins. Le PASS espère, grâce à la mobilisation de ses partenaires, l'engagement total de son équipe et un environnement sociopolitique régional stable, relever le défi de sa transformation au grand bénéfice des populations.

## Conflits d'intérêts

Les auteurs ne déclarent aucun conflit d'intérêts.

**Encadré n° 1**

**Ce qu'il faut retenir du PASS en quelques chiffres c'est:**

- 1 bureau à Abidjan;
- 6 relais permanents dans la zone UEMOA;
- + de 10 faîtières ouest-africaines et d'Afrique centrale appuyées et formées;
- + de 200 mutuelles bénéficiant d'accompagnement et de formation;
- + de 10 événements majeurs organisés en Afrique pour la promotion de la mutualité et de la protection sociale;
- + de 70 newsletters éditées et diffusées;
- + de 4 000 membres de la communauté PASS (Facebook – Twitter – LinkedIn).

**Encadré n° 2**

**Un programme transformé pour de nouveaux défis**

Dans sa logique de développement et comme l'avaient souhaité ses financeurs, le programme s'est aujourd'hui transformé en une structure pérenne et auto-financée. Au terme de sa « première vie » qui s'est achevée le 31 décembre 2020, le programme a commencé depuis août 2019 une transformation structurelle.

De programme financé, il devient, une société par actions simplifiées à but non lucratif, de droit ivoirien, portée par deux structures mutualistes françaises à savoir le Groupe Vyv et la Fédération Nationale de la Mutualité Française (FNMF).

Devenu Programme d'Appui aux Stratégies Sociales, la nouvelle version du PASS se présente comme un acteur majeur de développement. Un pôle d'expertise en santé et en protection sociale, qui se fixe pour mission de contribuer à l'amélioration du bien-être des populations sur le continent africain, en vue d'un meilleur accès à la santé et aux filets de protection sociale à des coûts abordables, ou sans contrepartie, lorsqu'il s'agit de communautés et de groupes en situation de vulnérabilité sévère. La structure se construit autour de:

- L'appui aux mécanismes solidaires de financement de la santé de type mutualiste (audit, études, conseil, formation)
- L'appui au renforcement des services de santé bâtis en lien avec les politiques publiques
- L'apport en expertise et en capacités opérationnelles en réponse à diverses problématiques ponctuelles de santé et de protection sociale

